# Environmental response strategies for the spatial distribution of seed plants in Gansu

**DOI:** 10.3389/fpls.2025.1526269

**Published:** 2025-02-17

**Authors:** Zizhen Li, Jia Wei, Weibo Du, Rong Huang, Lingling Song, Qing Tian, Xiaolei Zhou

**Affiliations:** ^1^ Forestry College, Gansu Agricultural University, Lanzhou, China; ^2^ Research Institute of Forestry, Chinese Academy of Forestry, Beijing, China; ^3^ Institute of Fruit and Floriculture Research, Gansu Academy of Agricultural Sciences, Lanzhou, China; ^4^ Gansu Academy of Agricultural Sciences, Lanzhou, China

**Keywords:** plant diversity, environmental response, joint species distribution models (JSDM), spatial distribution, Gansu

## Abstract

The interplay between plant diversity and environmental response strategies is crucial for ecosystem adaptability and stability. A central focus in modern ecology is elucidating how environmental factors shape plant diversity patterns and regulate species distributions across heterogeneous landscapes. This study employed Joint Species Distribution Model (JSDM) to quantitatively analyze the influence of environmental variables on plant spatial distributions in Gansu Province, China, while examining interspecies interactions under varying conditions. Results demonstrated that environmental factors explained 95.4% of the variance, highlighting their predominant role in determining plant distributions. Habitat type accounted for the largest share of variance (33.5%), followed by elevation (22.1%), mean annual temperature (20.3%), mean annual precipitation (15.1%), and solar radiation (4.4%). Species’ responses to environmental covariates were predominantly independent, with weak phylogenetic correlation (posterior mean: 0.17), reflecting limited ecological niche conservatism at the family level. Geographically, regions such as the northern Qilian foothills, Lanzhou-Baiyin wilderness, Loess Plateau, and Gannan Plateau exhibited negative correlations with most plant families, functioning as critical limiting or driving factors in spatial variability. Additionally, 33.7% of seed plant families showed negative correlations with light intensity, underscoring its role as a major limiting factor. Provincially, competition does not primarily constrain seed plant coexistence in Gansu. Regionally, however, pronounced differences in environmental responses were observed. In the northwest, solar radiation (37%) and precipitation (25%) were dominant drivers of plant distribution, while in the southeast, solar radiation (36.3%) and elevation (34.7%) were predominant. These findings underscore that species co-occurrence patterns are scale-dependent and influenced by regional resource availability. In resource-abundant southeastern areas, plant families displayed positive co-occurrence patterns indicative of mutualistic or symbiotic interactions, whereas resource-scarce northwestern areas experienced intensified negative co-occurrences due to heightened interspecific competition. This study highlights the critical role of environmental gradients in structuring seed plant distributions in Gansu, providing insights into the interaction of ecological adaptation and evolutionary history in shaping plant diversity. By identifying the drivers of plant distribution across heterogeneous environments, this research offers significant implications for biodiversity conservation and plant resource management strategies in Gansu Province, while contributing to a broader understanding of plant-environment dynamics in complex ecosystems.

## 1 Introduction

The variation in biodiversity and its spatial distribution results from a synergy of multiple mechanisms, including environmental heterogeneity, historical processes, biotic interactions, ecological drift, and niche differentiation. Understanding the factors that shape the spatial arrangement of species composition and abundance has long been a core topic in ecology and biogeography (Elo et al., 2021). In natural environments, species are intricately interrelated rather than existing in isolation. Characteristics such as species richness, abundance, ecological preferences, growth and reproductive strategies, and migratory behaviors are all influenced by interactions between biotic and abiotic factors (Pollock et al., 2014; Ovaskainen and Abrego, 2020). However, delineating the specific roles of these two ecological drivers in shaping population distribution remains a significant challenge in ecological research (Chen et al., 2023). A fundamental goal in ecology is to understand the determinants of species’ geographical distributions, where abiotic factors (e.g., temperature, precipitation) and biotic factors (e.g., species interactions, population dynamics) ultimately drive species distributions (Hutchinson, 1959). In contrast, community ecology often focuses on patterns of co-occurrence and community structure, with limited attention to co-occurrence phenomena that arise from species responding to shared environmental conditions (O’Reilly-Nugent et al., 2020).

Globally, ecosystems are facing unprecedented challenges due to climate change, habitat fragmentation, and biodiversity loss, all of which directly influence the spatial distribution of species (Gaston, 2000; Rahbek et al., 2019b; Mi et al., 2021). Climate change, in particular, is reshaping biodiversity patterns by altering species’ geographic ranges, phenologies, and interspecific interactions (Parmesan and Yohe, 2003; Bellard et al., 2012). For example, rising global temperatures have caused species to shift their distributions poleward or upward in elevation, disrupting local community compositions and ecosystem processes (Chen et al., 2011). Moreover, intensified desertification, particularly in arid and semi-arid regions, is exacerbating the loss of biodiversity, threatening fragile ecosystems worldwide (Huang et al., 2015). The same ecological challenges are faced in Gansu Province, where unique ecological and geographical conditions interact with environmental stressors to create a critical area for studying biodiversity patterns and ecological processes (Guo et al., 2023).

Gansu, located in the transitional zone between the Tibetan Plateau, Loess Plateau, and Inner Mongolia Plateau, represents a convergence of diverse ecosystems, ranging from arid deserts and semi-arid grasslands in the northwest to forested mountain regions in the southeast (Li et al., 2023). This geographic and ecological heterogeneity supports a wide range of plant species but also renders the region highly susceptible to global ecological challenges. In the northwest, areas like the Hexi Corridor are experiencing intensified desertification due to water scarcity, overgrazing, and climate variability, mirroring the global expansion of drylands under warming scenarios (Huang et al., 2015). These abiotic stressors are particularly severe in Gansu, where uneven precipitation, high solar radiation, and fragile soils combine to create an environment where plant survival and distribution are tightly constrained by environmental factors (Guo et al., 2023; Liu et al., 2024). Conversely, the southeastern regions of Gansu, such as the Qinling and Minshan Mountains, harbor biodiversity hotspots due to their favorable climatic conditions and rich vegetation cover (Pollock et al., 2014). These areas are highly sensitive to changes in temperature and precipitation, which are predicted to intensify under future climate scenarios (Masson-Delmotte et al., 2021). Shifts in species distributions driven by warming temperatures and altered precipitation patterns in these mountainous areas could disrupt mutualistic interactions (e.g., pollination and seed dispersal) and lead to a loss of biodiversity (Bascompte and Jordano, 2007). Furthermore, habitat fragmentation caused by land-use changes in these regions threatens to isolate plant populations, reducing genetic exchange and ecosystem resilience (Taubert et al., 2018).

At larger spatial scales (such as landscapes, regions, continents, and other broad areas), patterns of species composition and abundance are primarily attributed to climate and other abiotic factors, often overlooking the influence of biotic interactions (Wisz et al., 2013a). However, an increasing body of evidence suggests that biotic interactions, both within and between trophic levels, significantly shape these patterns beyond local scales, challenging traditional perspectives and offering new directions for research (Wisz et al., 2013a; Kissling and Schleuning, 2015). These findings highlight the importance of investigating how both positive and negative biotic interactions interact with environmental factors to shape species distributions, particularly in highly heterogeneous and vulnerable ecosystems like those in Gansu.

The application of statistical methods to observational data for assessing the influence of biotic interactions originated with studies examining patterns of species co-occurrence on islands (Kier et al., 2009). The main premise is that, relative to random co-occurrence predicted by null models, competitive interactions should result in a reduced co-occurrence among competing species (Gordijn and O’Connor, 2021). In recent years, growing interest in biotic interactions has spurred the development of numerous statistical approaches designed to investigate whether species co-occurrence frequencies are significantly lower than random expectations (indicating negative co-occurrence or segregation) or significantly higher than random expectations (indicating positive co-occurrence or aggregation) (Dormann et al., 2018). These techniques include single-species distribution models and dynamic occupancy models that account for the occupancy of other species (Wisz et al., 2013a), phylogenetic relationships (Paul et al., 2021), and ecological similarity (Beaudrot et al., 2019).

The Joint Species Distribution Model (JSDM) has emerged as a powerful tool for studying species distributions and community assembly processes (Pollock et al., 2014; Ovaskainen and Abrego, 2020). JSDMs simultaneously model species’ responses to environmental changes and community composition shifts, enabling the quantification of species associations and competitive effects, thereby addressing limitations of traditional community models (Wilkinson et al., 2021). Residual analysis in JSDMs allows for distinguishing positive, negative, or random species associations while controlling for environmental variables (Warton et al., 2015). Accounting for environmental variables is essential, as positive associations may reflect shared habitat preferences (species convergence), particularly among closely related species, while negative associations may result from differing habitat preferences (species differentiation) rather than direct competition (Elo et al., 2021).

A notable extension of JSDMs is the Hierarchical Modelling of Species Communities (HMSC), which employs a hierarchical Bayesian framework to integrate multiple levels of ecological complexity, such as phylogenetic relationships and biotic interactions (Farley et al., 2018; Tikhonov et al., 2020). HMSC improves upon traditional JSDMs by distinguishing biotic interactions from shared environmental responses, providing a more robust framework for analyzing ecological data. Additionally, HMSC’s incorporation of prior ecological knowledge and flexibility in accommodating various data types enhances its utility in understanding species interactions and community dynamics (Tikhonov et al., 2020). Despite being more computationally intensive, advances in methods such as the latent variable model (LVM) proposed by Warton et al. (2015) have mitigated these challenges, enabling efficient analysis of large and complex datasets.

JSDMs, particularly in their hierarchical forms, demonstrate significant advantages over traditional species distribution models by improving predictive accuracy, especially when species data are sparse (Tikhonov et al., 2017). These models not only distinguish environmental factors from species interactions but also provide a comprehensive framework for integrating ecological information, making them highly applicable for modern ecological research (Ovaskainen et al., 2017a; Chen et al., 2023).

This study aims to explore how environmental factors and interspecific interactions influence the community structure and spatial distribution of seed plants in the heterogeneous landscapes of Gansu Province. Using the HMSC, we systematically analyzed the relative contributions of environmental gradients, spatial factors, and phylogenetic relationships in shaping plant community patterns. By integrating species distribution data, environmental variables, and phylogenetic information with advanced statistical methods, this study seeks to uncover the patterns by which plant communities respond to environmental changes and to elucidate the ecological processes underlying interspecific interactions in regions with significant environmental heterogeneity. The primary aim of this research is to provide reliable predictions of plant species habitat distribution and to deepen our understanding of the interactions between seed plants and their environment in Gansu. Specifically, the study aims to reveal how abiotic factors such as habitat types, elevation, and climate interact with biotic processes, including competition and facilitation, to shape regional biodiversity patterns. By identifying key drivers of plant community structure and key constraints to species persistence, an effective scientific basis for biodiversity conservation and sustainable management in Gansu Province will be provided. In addition, the findings will provide valuable insights for biodiversity research in other arid and semi-arid regions with similarly complex ecological conditions.

## 2 Materials and methods

### 2.1 Study area

**Figure 1:**
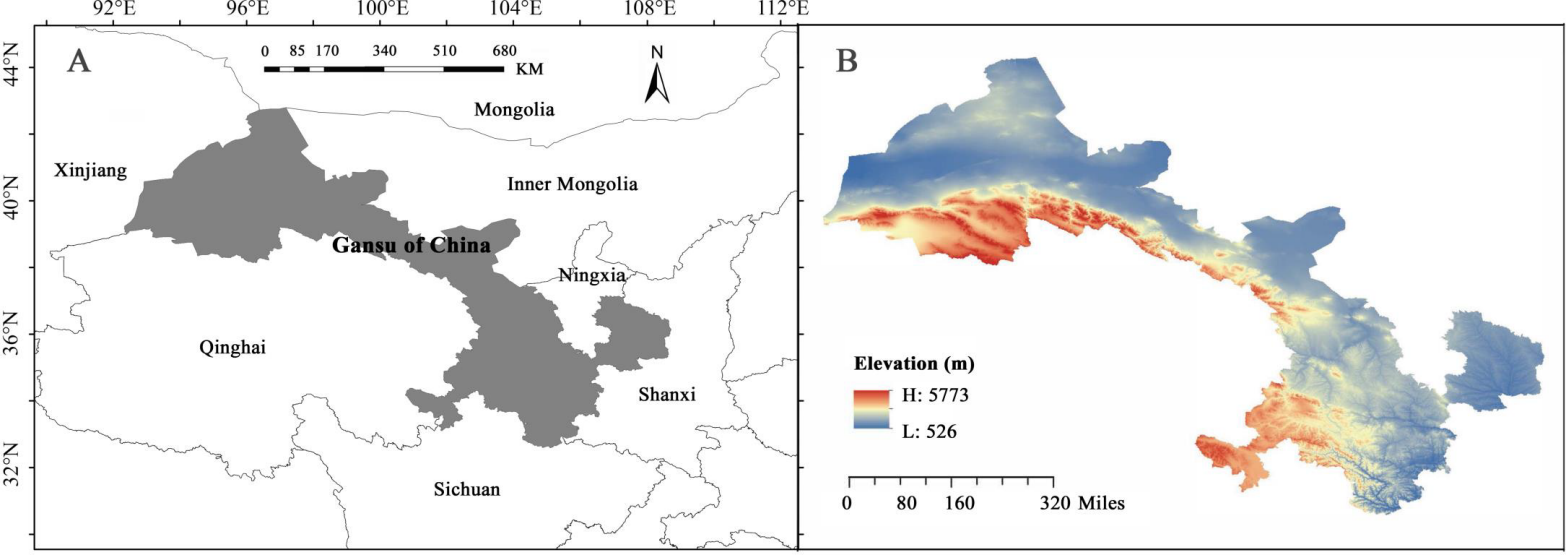
Geographical location **(A)** and elevation distribution **(B)** in Gansu Province.

Gansu Province, a narrow, elongated region in northwestern China, covers an area of 453,700 square kilometers, located between latitudes 32°11′ N - 42°57′ N and longitudes 92°13′ E - 108°46′ E, with elevations ranging from 526 to 5773 meters (Zhang and Zhu, 2020) (**Figure 1**). Positioned at the intersection of the Loess Plateau, the Inner Mongolian Plateau, and the Qinghai-Tibet Plateau, Gansu exhibits highly diverse natural geography and climate (Mao et al., 2020). According to Rivas-Martínez’s global bioclimatic classification, the region spans four major climatic types, including warm temperate continental and cold temperate continental climates (Rivas-Martínez et al., 2011), and serves as a critical water conservation and supply area for the upper reaches of the Yangtze and Yellow Rivers (Hu, 2024). The geological, topographic, and climatic diversity of Gansu has been instrumental in shaping the region’s unique floristic composition (Hu, 2024).

Gansu Province is characterized by several major mountain ranges, including the Qilian, Qinling, and Liupan Mountains, which serve as both geographical and ecological boundaries. Many county boundaries align with the ridgelines of these mountains, establishing natural and administrative borders (Zhang, et al., 1936). Key rivers such as the Yellow, Tao, and Wei Rivers in eastern Gansu, as well as the Shiyang, Heihe, and Shule Rivers in the Hexi Corridor, further define the province’s complex hydrological system. These rivers frequently act as natural boundaries between counties and often traverse administrative regions, influencing county demarcation (Gansu Local History Committee, 2018). Due to the limitations in the accuracy of species distribution data, plant species information in Gansu can only be reliably resolved at the county level. The administrative boundaries within Gansu closely follow natural features like mountain ridges and river paths, making counties the most suitable minimum units for this study. This approach aligns with the geographical distribution characteristics of plants in Gansu and enhances the precision of ecological and biodiversity analyses. To ensure geographical continuity and integrity for the analysis of species composition and phylogenetic relationships, adjustments were made to merge or consolidate adjacent areas or fragmented territories within counties.

### 2.2 Construction of plant dataset and environmental dataset

From May 2021 to January 2022, we compiled records of seed plants in Gansu Province. The dataset was developed using resources such as the Flora of Gansu (Gansu Flora Committee, 2005), Lanzhou Flora (Sun, 1962), Flora of China (Committee of the Flora of China, 2004), Flora of Chinese Deserts (Liu, 1985), Flora of the Loess Plateau (Fu, 2000), Flora of Ziwu Mountain Woody Plants (Liu, 1998), Flora of Kongtong Mountain (Gao, 1998), Flora of Xiaolong Mountain Higher Plants in Gansu Province (An, 2002), Illustrated Guide to Gannan Trees (Feng, 1994), Gansu Medicinal Plant Resources (Zhao, 2004), and Vegetation of Gansu (Huang, 1997), along with relevant journal articles documenting all available plant records in Gansu (Appendix 1). To supplement and refine the dataset, we consulted authoritative databases, including the Chinese Virtual Herbarium (www.cfh.ac.cn), Chinese Digital Herbarium (www.cvh.ac.cn), National Specimen Information Infrastructure (www.nsii.org.cn), Global Biodiversity Information Facility (GBIF) (www.gbif.org), Catalogue of Life China (www.sp2000.org.cn), Catalogue of Life (www.catalogueoflife.org), and the Flora of China (www.iplant.cn). These platforms provided additional plant records to enhance the completeness and accuracy of the dataset. The data were purged of duplicates and records that lacked reliable georeferencing to the level of the county. Excluding non-native species, the final matrix consisted of 5244 records belonging to 1131 genera and 170 families. The names of all species were standardized using the R (R Core Team, 2017) software package ‘plantlist’ (Zhang, 2019), with the order of families based on the nomenclature of Angiosperm Phylogeny Group IV (Group, 2016) and on the most recent gymnosperm classification system (Yang et al., 2022). For this analysis, the study area was divided into 80 county-level geographical units (**Supplementary Figure S1** and **Supplementary Table S1**). The presence or absence of seed plants in each county was determined by extracting distribution information from the county grid and constructing a presence-absence matrix (**Supplementary Table S2**).

In this study, we constructed an environmental variable dataset to investigate the adaptability of plant spatial distribution to environmental factors (**Table 1**). First, we utilized three climate variables: mean annual temperature (MAT), mean annual precipitation (MAP), and mean annual solar radiation (Srad), sourced from WorldClim (http://www.worldclim.org) at a resolution of 10 km × 10 km for the period from 1970 to 2000. This timeframe was chosen as it provides a robust long-term average of climate conditions, minimizing the influence of interannual variability and representing a stable baseline for analyzing plant-environment relationships. Furthermore, it is widely regarded as a pre-global climate change period, capturing the natural conditions to which plants have historically adapted, and has been extensively used in similar ecological and biogeographical studies (Thomas et al., 2004; Hijmans et al., 2005; Araújo and Rahbek, 2006; Elith et al., 2006; Karger et al., 2017). Next, we used the average elevation from Digital Elevation Model (DEM) data at a 30 m resolution to represent the mean altitude of the county grid units. Finally, we incorporated nine floristic regions identified in our previous study as habitat types: I: the northern foothills of the Qilian Mountains; II: the hinterland of the Hexi Corridor; III: the Lanzhou-Baiyin wilderness region; IV: the Loess Plateau in the central region; V: the Loess Plateau in the east region; VI: the western Qinling Mountains; VII: the transitional zone from Gannan Plateau to Longnan Mountainous Region; VIII: the Gannan Plateau; and IX: the Longnan Mountainous Region (**Supplementary Figure S3**) (Li et al., 2023). Climate data for the Gansu region were extracted using ArcGIS 10.8. To evaluate the shared variance among predictors, we use the cor function in R to compute the correlation matrix for the predictors (MAT, MAP, Elev, Srad).

**Table 1:** Environmental factors.

Variable	Abbreviation
Mean annual temperature (°C)	MAT
Mean annual precipitation (mm)	MAP
Elevation (m)	Elev
Solar radiation (kJ m^-2^ day^-1^)	Srad
Habitats	Hab

### 2.3 Phylogenetic analysis

This study constructed the phylogenetic tree for seed plants in Gansu based on the mega-tree created by Jin and Qian (2019) using the Phylomatic framework. This mega-tree integrates various phylogenetic insights: it includes the evolutionary tree of angiosperms calibrated with paleontological and fossil data, derived using penalized likelihood (Smith and O’Meara, 2012) and Bayesian relaxed clock methods (Drummond et al., 2006; Drummond and Rambaut, 2007) as per Magallón et al. (2015). It also incorporates a dated phylogenetic tree for seed plants (GenBank taxa with a backbone from the Open Tree of Life project: GBOTB) constructed by Smith and Brown (2018) using genetic and systematic data from the GenBank database (https://tree.opentreeoflife.org), along with phylogenetic branches for ferns from Zanne et al. (2014). This mega-tree comprises 10,587 genera and 74,533 species of vascular plants, covering all 479 families of vascular plant globally (Jin and Qian, 2019).

Using this mega-tree, the families identified within the study region were integrated into the existing backbone phylogeny through R software with the ‘V.PhyloMaker2’ package (Jin and Qian, 2019) to generate a phylogenetic tree at the family level. Families absent from the mega-tree were assigned to their closest relatives to complete the phylogeny (Jin and Qian, 2019). Due to limited availability of comprehensive time-calibrated phylogenies at the family and genus levels, we applied a similar method to previous studies (Hardy et al., 2012), treating unassigned families as multiple families within their respective orders. The constructed phylogenetic tree was then visualized using the online software Interactive Tree of Life (ITOL) (https://itol.embl.de) (**Supplementary Figure S2**).

### 2.4 JSDM model construction

In this study, we employed the Hierarchical Modeling of Species Communities (HMSC) framework to conduct a comprehensive analysis of environmental responses and interspecies relationships among plant communities in Gansu. Initially, we constructed a presence-absence matrix (*Y*) utilizing plant distribution data across 80 county-level grid units, ensuring accurate representation of species occurrences within each spatial unit. For the fixed effects component, we developed a predictor matrix (*X*) encompassing five key environmental variables: mean annual temperature (MAT), mean annual precipitation (MAP), mean elevation (Elev), mean annual solar radiation (Srad), and habitat type (Hab). These variables were selected based on their ecological relevance and availability at the county level, acknowledging the limitation of lacking fine-scale climate data by relying on broad-scale climatic indicators (Warton et al., 2015; Ovaskainen et al., 2016a).

During the model construction phase, each sampling unit was assigned a random effect to account for spatial heterogeneity, with the number of latent variables constrained to two to efficiently estimate residual associations among species. These residual associations, captured within the random effects component, reflect intra-species variability and potential shifts in interspecies interactions, thereby elucidating underlying community dynamics (Ovaskainen et al., 2016b). Additionally, we incorporated a species phylogenetic tree into the model, integrating phylogenetic relationships with geographical coordinates of the county-level grids to account for spatial variation and phylogenetic dependencies within the community structure (Tikhonov et al., 2020).

Given the computational challenges associated with the variance-covariance matrix, which scales quadratically with the number of species, we implemented a Latent Variable Model (LVM) (Warton et al., 2015; Ovaskainen et al., 2016a). The LVM approach facilitated the handling of datasets encompassing hundreds of species by effectively managing multilevel spatial-temporal structures and various sources of uncertainty, including unobserved environmental variables. The latent variable model was formalized through the equation:


$$g(m_{ij})=X_{i}\beta_{j}+Z_{i}\lambda_{j}$$

where *g* represents the link function, *m_ij_* denotes the abundance or probability of occurrence for species *j* at sampling point *i*, *X* comprises the observed environmental predictors, and *Z* signifies the latent variables. The coefficients *β* and *λ* quantify species’ responses to environmental and latent variables, respectively. The random effects component, *Zλ*, was estimated directly from the observed data, allowing for the simultaneous estimation of fixed and random effects and thereby capturing complex species-environment interactions and unobserved factors influencing community structure.

Model fitting was conducted within a Bayesian framework using Markov Chain Monte Carlo (MCMC) sampling, implemented via the ‘HMSC’ package (Tikhonov et al., 2020). The MCMC sampling scheme was carefully controlled by adjusting key parameters, including the number of posterior samples, thinning interval number of steps between retained samples), burn-in length (transient phase), and the number of chains. Additionally, the parameter ‘adaptNf’ was used to control the number of iterations during which the number of latent factors was adapted, ensuring optimal model flexibility. Two independent MCMC chains were initialized, each consisting of 7500 iterations. The first 2500 iterations of each chain were discarded as burn-in to eliminate transient effects and allow the chains to converge to a stationary distribution. For the remaining 5000 iterations, thinning was applied by retaining every 100th sample, reducing autocorrelation among successive samples. This resulted in 1000 posterior samples per chain and a combined total of 2000 posterior samples across both chains.

To evaluate the performance of the sampling procedure, chain mixing and convergence were assessed using diagnostic tools from the ‘coda’ package. The posterior distributions were converted into coda-format using the function ‘convertToCodaObject’, which facilitates the application of standard convergence diagnostics. Convergence of the MCMC chains was assessed using two key diagnostics: the effective sample size (ess) and the potential scale reduction factor (psrf). ess was calculated to evaluate the degree of autocorrelation among successive samples, with higher ess values indicating lower autocorrelation and greater efficiency of the sampling process (Gelman and Rubin, 1992). psrf was employed to compare the variance within each chain to the variance between chains, with values approaching 1 signifying satisfactory convergence (Brooks and Gelman, 1998). A comprehensive evaluation of model convergence was conducted by integrating the adjusted ess and psrf values, ensuring the reliability and stability of the posterior estimates.

To evaluate the explanatory and predictive performance of the model, we implemented a two-fold cross-validation strategy. Model performance was quantified using species-specific Area Under the Curve (AUC) metrics and Tjur’s *R²* statistic, which measures the proportion of variance in the data explained by the model (Pearce and Ferrier, 2000; Tjur, 2009). The cross-validation process involved partitioning the data into training and testing sets, fitting the model on the training data, and subsequently predicting on the testing data to assess the model’s predictive accuracy and mitigate overfitting. We assumed uniform residual variation across all plant species to further enhance the robustness of the predictive assessments. The *R²* values, ranging from 0 to 1, facilitated comparative evaluations of model performance, with higher values indicating superior explanatory and predictive capabilities (Xu et al., 2023).

Following model selection, the optimal HMSC model was utilized to elucidate plant responses to environmental factors and interspecies relationships within the Gansu region. Variance partitioning was conducted to quantify the proportion of variation attributable to each environmental variable, employing the ‘computeVariancePartitioning’ function within the ‘Hmsc’ package (Tikhonov et al., 2020). Visualization of the variance partitioning results was achieved using the ‘plotVariancePartitioning’ function, providing clear graphical representations of the relative importance of each predictor. Species-specific responses to environmental variables were interpreted through the regression coefficients *β*, indicating the direction and strength of correlations between plant species and environmental factors. Interspecies relationships were examined via the latent factor *Ω* parameter, which revealed the variability in correlations among plant species. The regression coefficients were visualized using heatmaps to facilitate the identification of patterns, while the *Ω* parameter was depicted using the ‘corrplot’ package (Wei and Simko, 2021) to illustrate the network of interspecies associations. All model construction, fitting, and subsequent analyses were performed using R 4.3.2, ensuring reproducibility and consistency in the analytical procedures.

## 3 Results

### 3.1 Correlation of environmental factors

**Figure 2:**
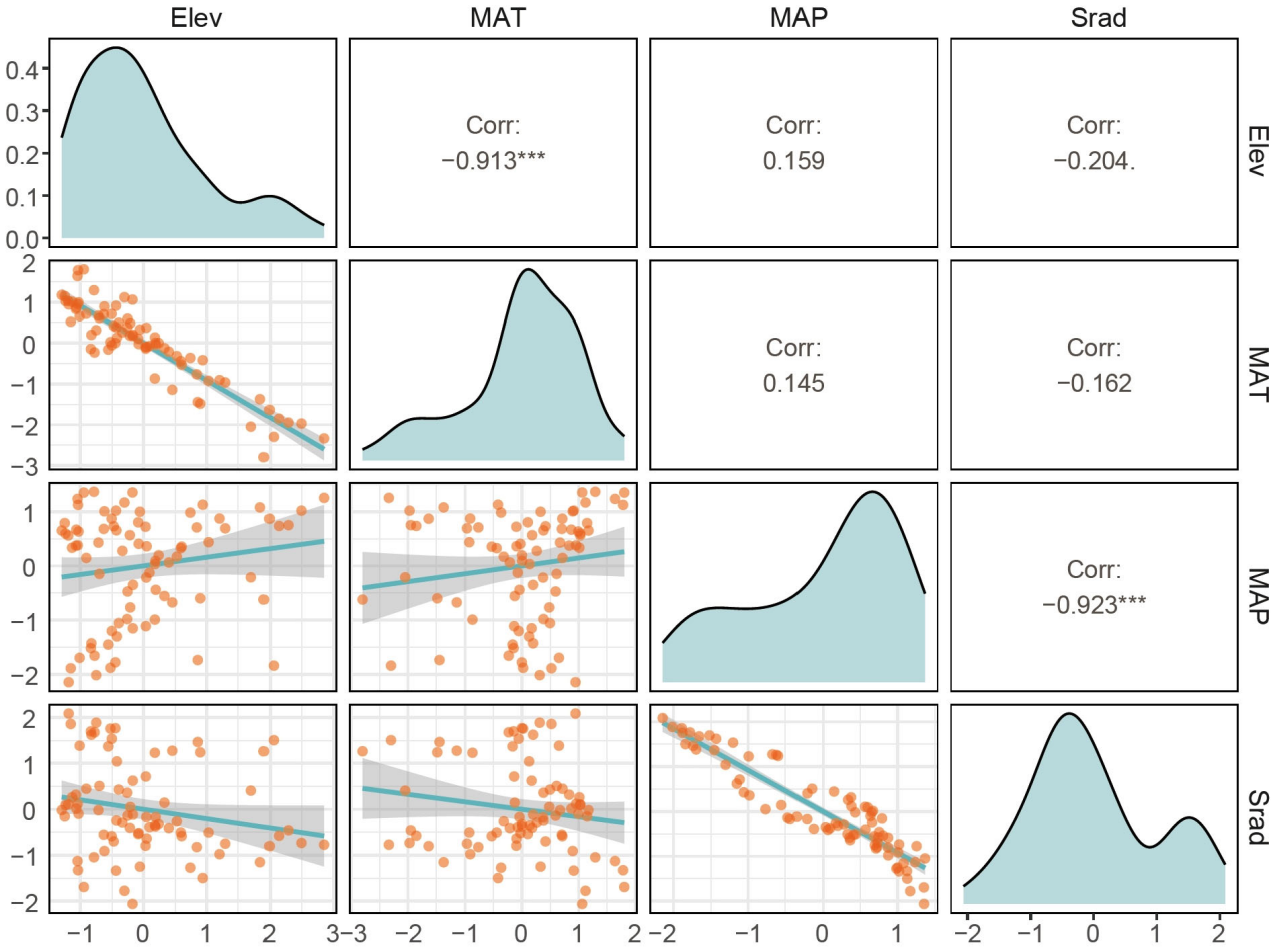
Correlation of environmental factors in Gansu. The upper triangle displays the correlation coefficients, the diagonal shows density plots, and the lower triangle presents scatter plots. The density and scatter plots illustrate the specific distribution and trends of each environmental variable. The larger the correlation coefficient, the larger the corresponding font size, and vice versa. ***p<0.001, **p<0.01, *p<0.05. Elev, MAT, MAP and Srad stand for elevation, mean annual temperature, mean annual precipitation and mean annual solar radiation, respectively.

The correlation analysis of environmental factors at the county level in Gansu Province (**Figure 2**) indicates that MAT and Elev are significantly negatively correlated (*p* < 0.001) with a correlation coefficient of -0.913. MAP shows a nonsignificant positive correlation with Elev and MAT (*p* > 0.05), with correlation coefficients of 0.159 and 0.145, respectively. However, MAP is significantly negatively correlated with Srad (*p* < 0.001) with a correlation coefficient of -0.923. Srad exhibits a nonsignificant negative correlation with Elev and MAT (*p* > 0.05), with correlation coefficients of -0.204 and -0.162, respectively.

### 3.2 HMSC diagnostics and model performance

**Figure 3:**
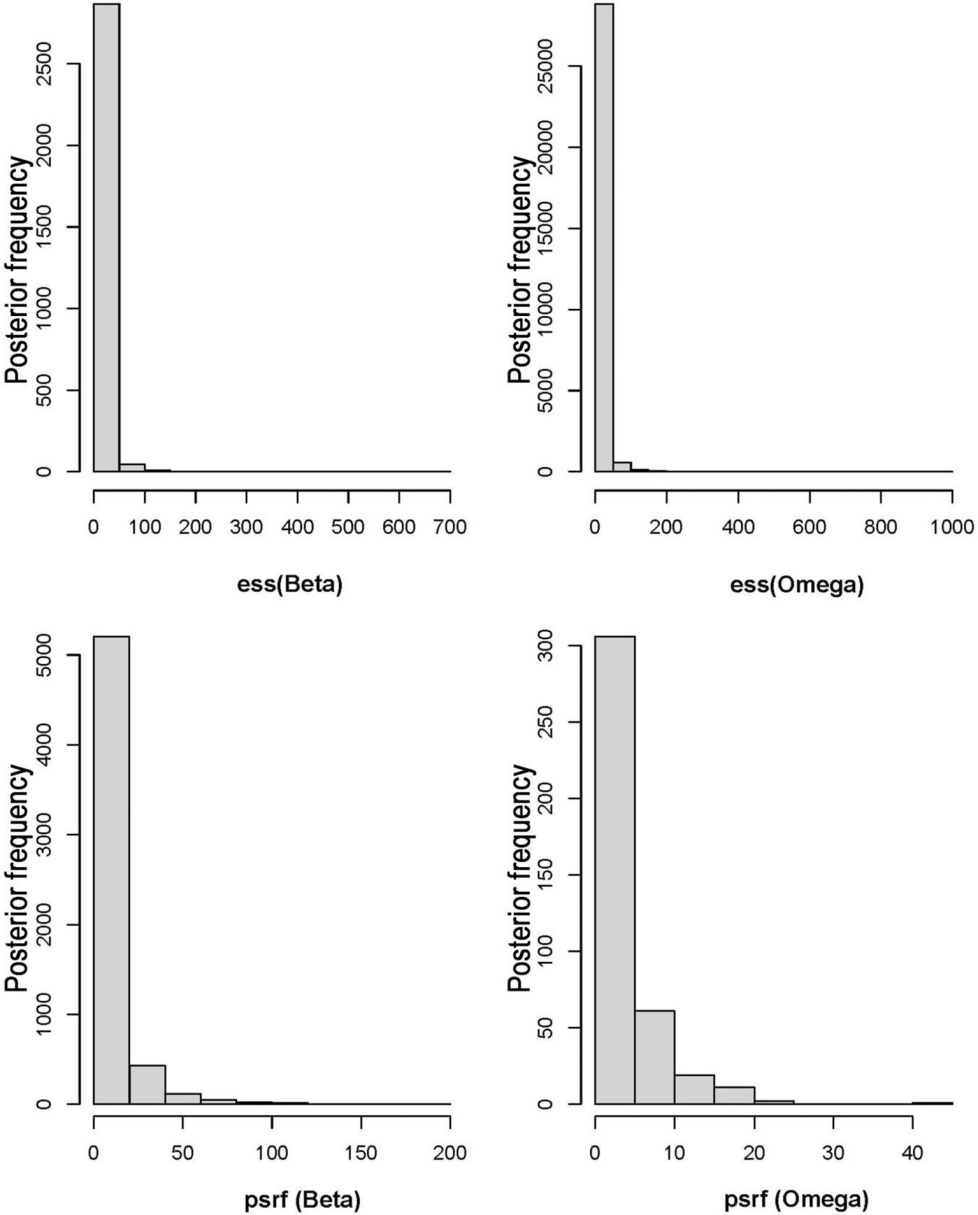
Effective sample sizes (ess) and potential scale reduction factors (psrf) for Beta and Omega parameters.

**Figure 4:**
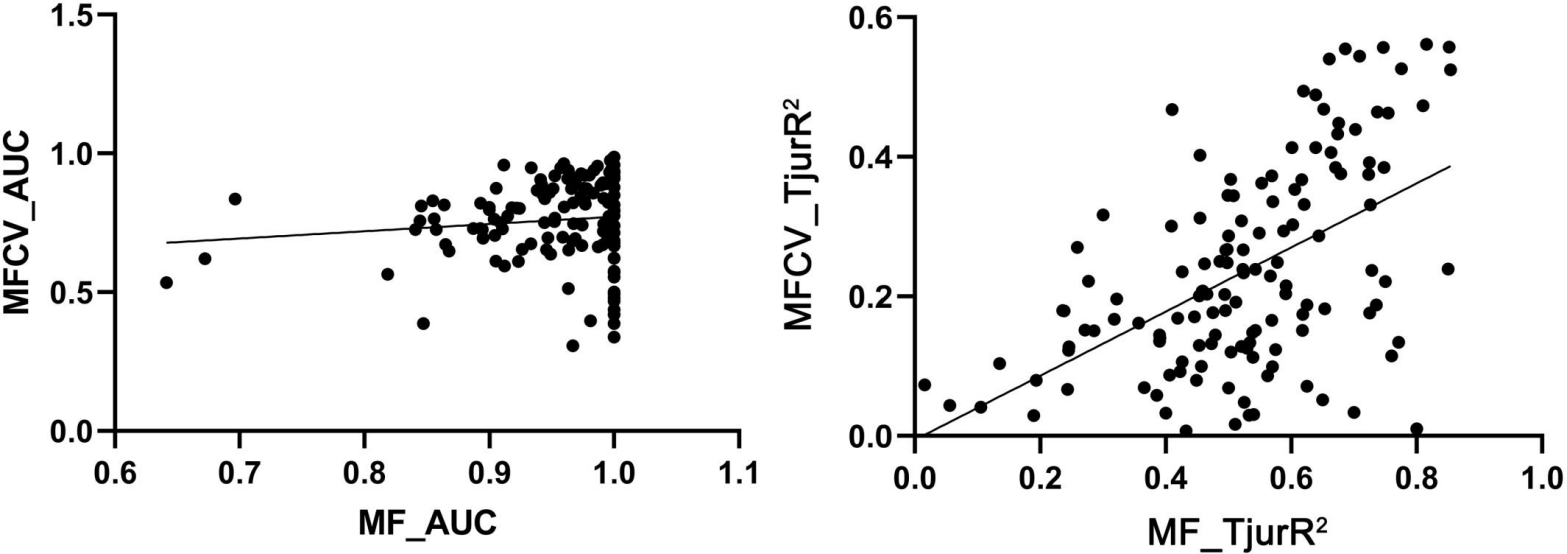
Explanatory and predictive power of models.

The HMSC diagnostics indicated satisfactory MCMC convergence. For the beta parameters (representing species responses to environmental variables) and Omega parameters (representing species associations at the site level), the effective number of posterior samples was close to the expected 2500, demonstrating the efficiency of the sampling process and low autocorrelation between successive samples. Additionally, the sampling quality of the Omega parameter was also high. Further analysis showed that the potential scale reduction factors (psrf) for both Beta and Omega parameters were close to 1, indicating minimal variability in the sampled results and high consistency across posterior distributions generated by the two chains, verifying the model’s stability and reliability (**Figure 3**). The model fit (Model Fit, MF) and cross-validated predictive performance (Model Fit Cross-Validation, MFCV) were also satisfactory. The average AUC values for explanatory and predictive power were 0.95 and 0.76, respectively, while the average Tjur’s R² values were 0.53 for explanatory power and 0.42 for predictive power (**Figure 4**).

### 3.2 Response of species distribution to environmental variables

**Figure 5:**
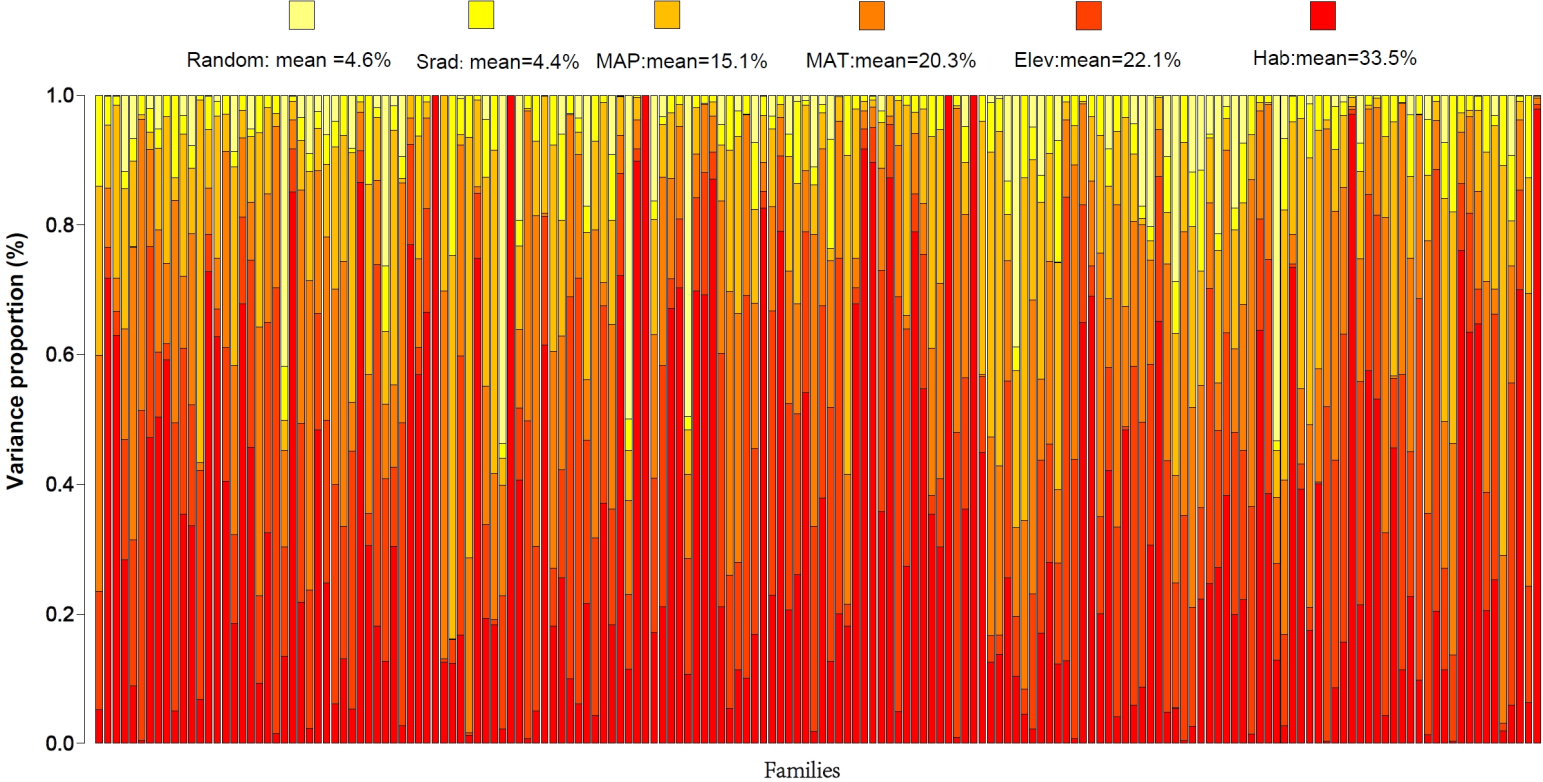
Environmental explanatory rate of variance partitioning in the spatial distribution of Gansu plants.

The fixed and random effects varied in their influence on the spatial distribution of plant species in Gansu Province. Variance partitioning of environmental factors revealed the proportion of variance explained by each factor (**Figure 5**). For plant species distribution in Gansu, the JSDM model’s fixed effect variance partitioning (VP) averaged 95.4%, while the relative proportion of explainable variance by random effects was comparatively low (V_Prandom_ = 4.6%). The contribution of each environmental variable to species distribution, in descending order, was as follows: habitat type (VP_Hab_ = 33.5%) > elevation (VP_Elev_ = 22.1%) > mean annual temperature (VP_MAT_ = 20.3%) > mean annual precipitation (VP_MAP_ = 15.1%) > solar radiation (VP_Srad_ = 4.4%).

Species distribution patterns generally exhibit spatial characteristics and are closely tied to habitat types. Regarding species tolerance and preference, habitat type was the most influential factor on plant distribution, affecting 27.9% of families (48 families) with a variance partitioning (VP) greater than 50.0%. Among these, 15 families, such as Cynomoriaceae, Nyctaginaceae, Frankeniaceae, and Cistaceae, showed particularly high sensitivity to habitat type (VP > 80.0%). Elevation primarily influenced the distribution of 14 families, such as Pentaphylacaceae, Begoniaceae, Theaceae, Ephedraceae, and Melastomataceae, with VP values exceeding 50.0%. Pentaphylacaceae, in particular, was most affected by elevation (VP = 71.5%). Mean annual temperature significantly impacted families like Colchicaceae, Ruppiaceae, and Nelumbonaceae (VP > 50.0%), while mean annual precipitation most prominently influenced Coriariaceae, Verbenaceae, Commelinaceae, and Aquifoliaceae (VP > 50.0%). When considering all observed environmental variables together, solar radiation had a comparatively limited effect on plant distribution. For random effects, the most affected families were Cupressaceae and Salicaceae, with VP values of 53.6% and 53.3%, respectively.

**Figure 6:**
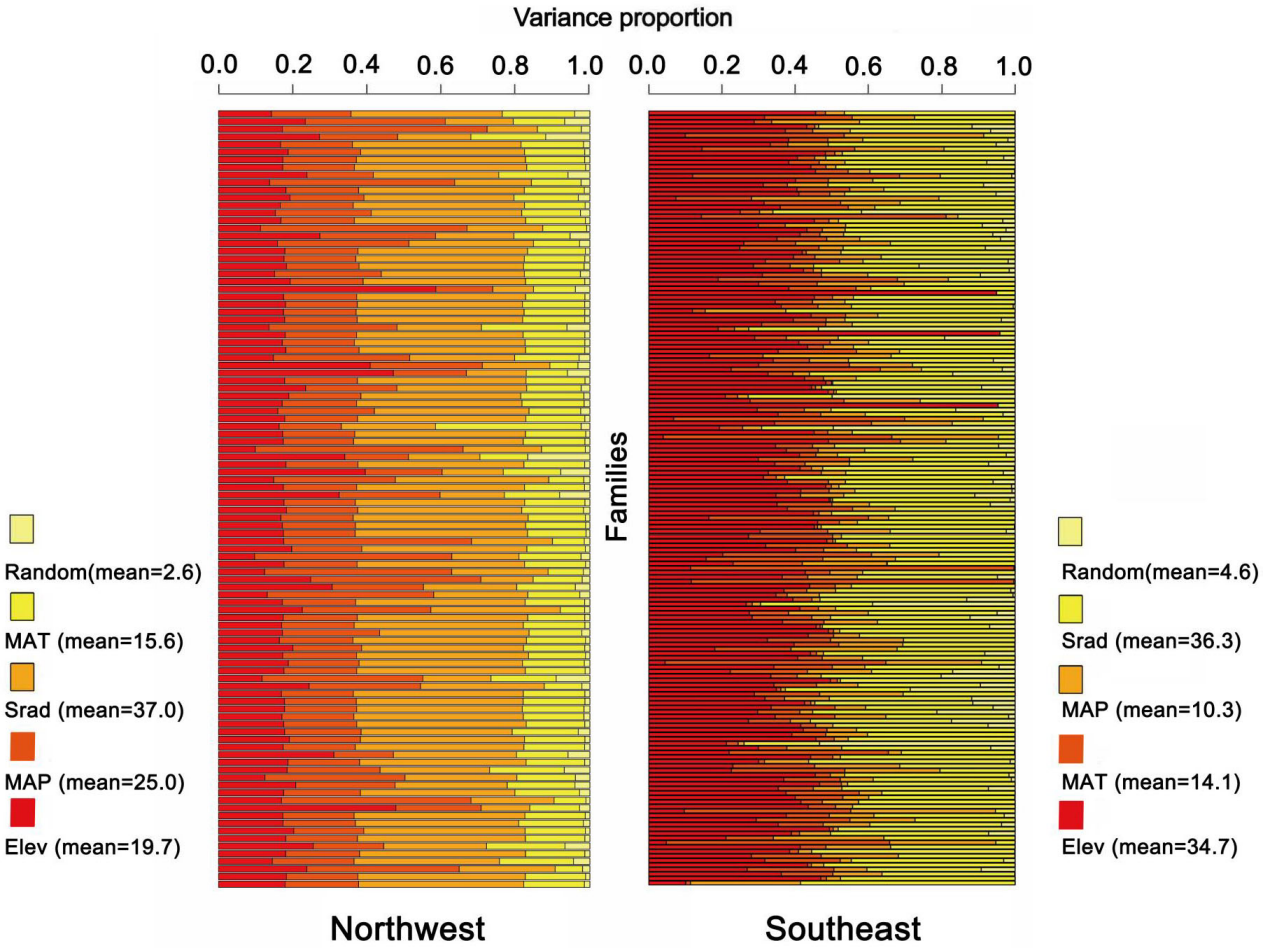
Environmental interpretations of plant distribution in two major zones in northwest and southeast Gansu.

Our previous study showed that the spatial distribution of plants in Gansu exhibits obvious east-west differences (Li et al., 2023) (**Supplementary Figure S3**), suggesting that there are significant differences in the preferences and sensitivities of plants to distributed environmental factors between the flora regions of northwestern and southeastern Gansu. To further investigate the environmental drivers of floristic differentiation, we constructed JSDM models at the family level for 102 families in the northwest region and 170 families in the southeast region. The results indicate that (**Figure 6**), for the northwest region (102 families), the influence of environmental variables ranked as follows: solar radiation (VP_Srad_ = 37%) > mean annual precipitation (VP_MAP_ = 25%) > elevation (VP_Elev_ = 19.7%) > mean annual temperature (VP_MAT_ = 15.6%) > random effects (VP_Random_ = 2.6%). Clearly, solar radiation and precipitation are the primary environmental factors affecting the northwest floristic region. In the southeast region (170 families), the variables ranked in order of influence as follows: solar radiation (VP_Srad_ = 36.3%) > elevation (VP_Elev_ = 34.7%) > mean annual temperature (VP_MAT_ = 14.1%) > mean annual precipitation (VP_MAP_ = 10.3%) > random effects (VP_Random_ = 4.6%). Here, solar radiation and elevation emerged as the dominant factors influencing plant distribution in the southeast floristic region. These results highlight that while solar radiation plays a major role in both regions, precipitation and elevation are more region-specific, underscoring their differential impacts on plant distribution across the northwest and southeast floristic zones.

**Figure 7:**
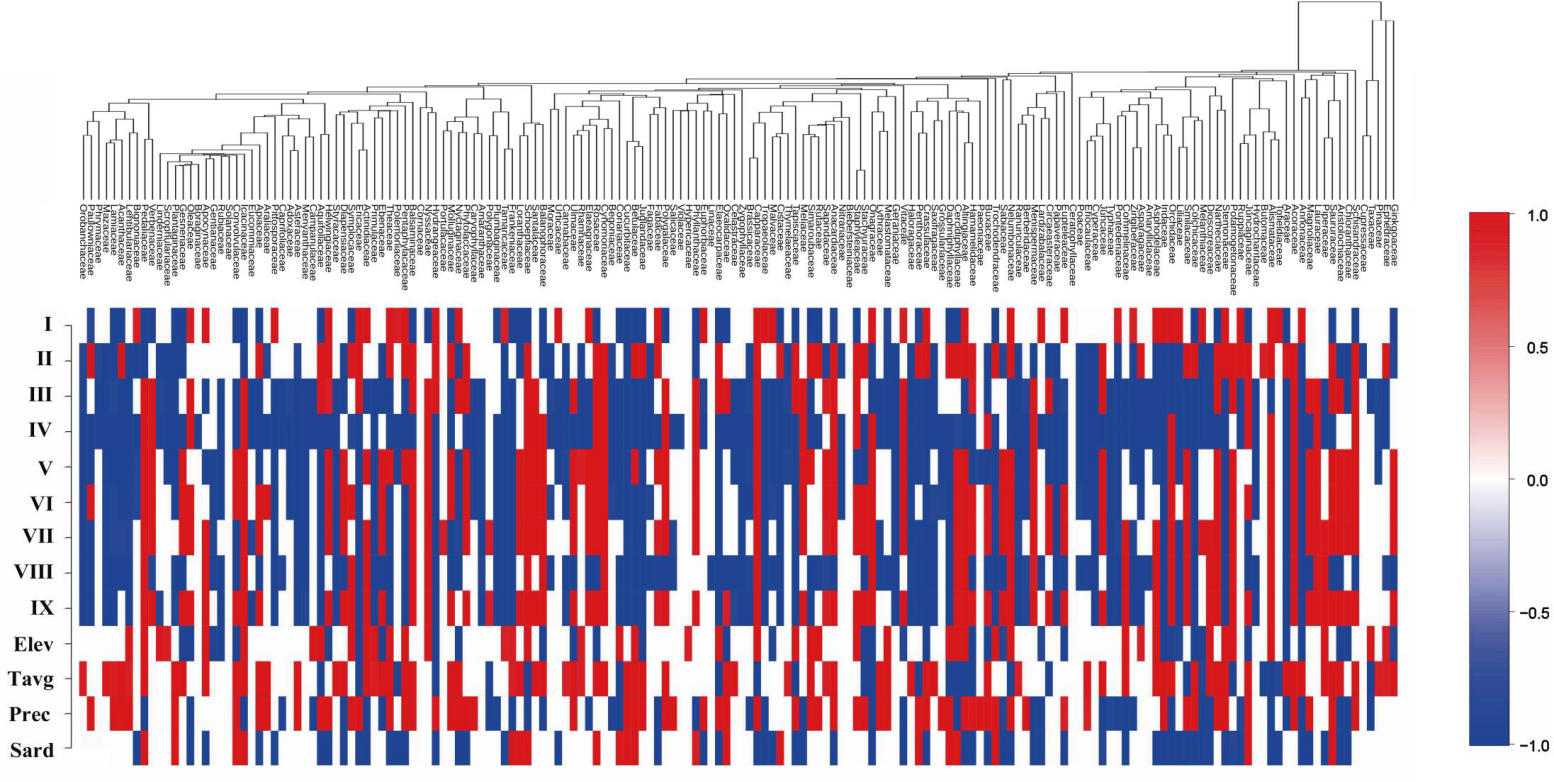
Significance of species response to environmental factors. I - IX are the nine flora types (I: the northern foothills of the Qilian Mountains; II: the hinterland of the Hexi Corridor; III: the Lanzhou–Baiyin wilderness region; IV: the Loess Plateau in the central region; V: the Loess Plateau in the east region; VI: the western Qinling Mountains; VII: the transitional zone from Gannan Plateau to Longnan Mountainous Region. VIII: the Gannan Plateau; and IX: the Longnan Mountainous Region), and Elev, MAT, MAP, and Srad are the β-parameters corresponding to the environmental variables mean elevation, mean annual temperature, mean annual precipitation, and mean annual solar intensity, respectively.

A heatmap was used to illustrate plant families’ sensitivity to habitat and environmental factors (**Figure 7**). The results indicate that the overall response of plant families in Gansu to environmental covariates is relatively independent and not strongly phylogenetically structured, as reflected by a posterior mean phylogenetic parameter (β) of 0.17, which quantifies the degree to which phylogeny explains variation in responses to environmental factors (with a 97.5% confidence interval). However, certain phylogenetically related groups show similar responses to environmental covariates. For example, many families within the Lamiales order, such as Orobanchaceae, Phrymaceae, Mazaceae, Lamiaceae, and Bignoniaceae, demonstrate negative correlations with floristic regions other than region I. These families are particularly sensitive to temperature and precipitation, preferring cooler, water-rich environments like those found in the Qilian Mountains in Gansu (**Figure 7**). Spatially, regions I, III, IV, V, and VIII exhibit negative correlations with the occurrence of most families (27.6%, 55.9%, 69.4%, 45.9%, 58.2%, respectively), suggesting that the unique geographic settings of the Qilian Mountains, Loess Plateau, and Gannan Plateau may serve as major constraints or drivers on plant distribution across much of Gansu. The analysis further reveals that temperature and precipitation are positive influences on the survival of many plant species in Gansu, with 41.3% and 35.5% of families showing positive correlations with temperature and precipitation, respectively. Moreover, solar radiation emerged as the most restrictive factor on plant distribution and dispersal, with 33.7% of families showing a negative correlation with solar intensity. Families such as Oleaceae, Helwingiaceae, Aquifoliaceae, and Tropaeolaceae are particularly light-sensitive, indicating a preference for environments with moderate sunlight. Overall, the model effectively quantifies plant responses to environmental covariates, providing clear insights into environmental preferences critical for plant survival in Gansu.

### 3.3 Interspecific relationship

**Figure 8:**
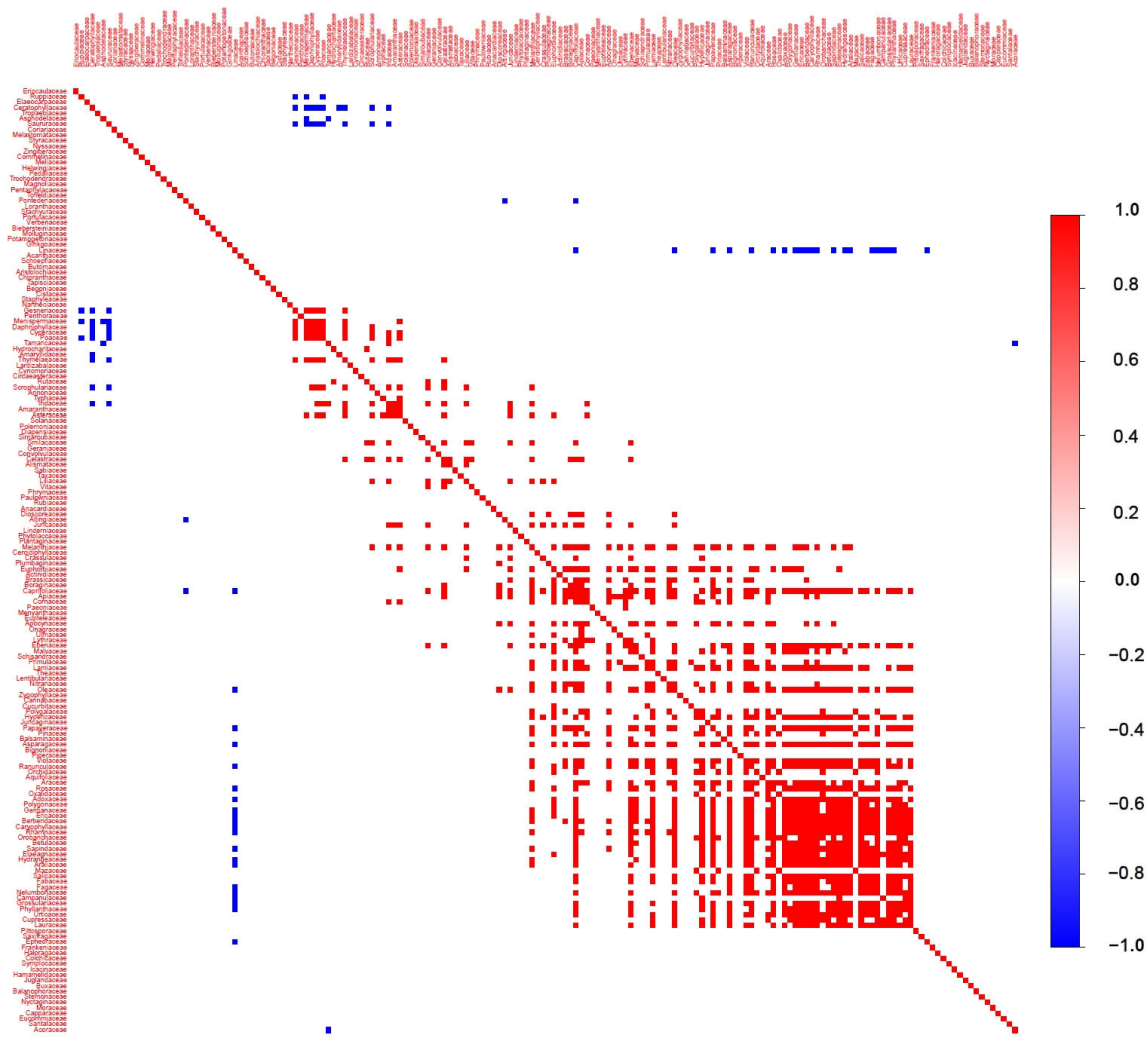
Associations among plant species in Gansu. Association is the posterior probability that species co-occur (co-occurring between species on spatial or temporal scales more or less frequently than chance after considering their environmental ecological niche) with statistical support ≥ 95% after taking into account their environmental response.

After controlling for environmental factors, we conducted an in-depth analysis of interspecies associations among plant families in Gansu (**Figure 8**). The results suggest that interspecies interactions have a relatively low overall influence on species distribution at the family level, though they play a crucial role for certain families. Specifically, a few families, including Linaceae, Ceratophyllaceae, and Saururaceae, show significant negative correlations with other families. Most other families either exhibit no significant associations or significant positive correlations with other families. Although the effects of interspecific associations are usually smaller than those of environmental factors, they may still play a key role in shaping the distribution patterns of certain plant families in Gansu.

**Figure 9:**
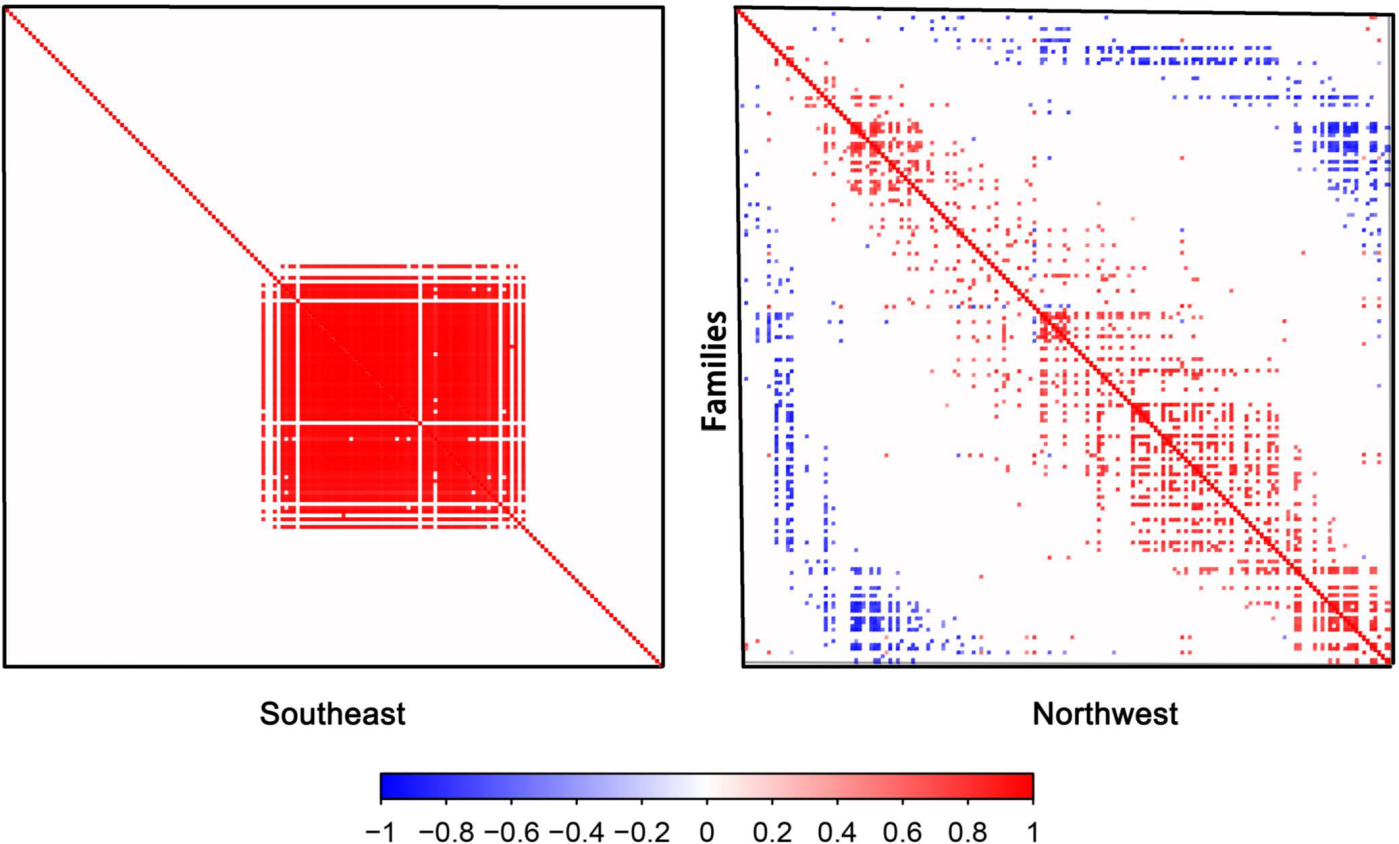
Plant species associations between the southeastern and northwestern parts of Gansu.

Our previous research indicated that the spatial distribution of plants in Gansu displays significant differences between the east and west (Li et al., 2023). Consistent with our previous study, the present study further demonstrated that the residual co-occurrence rates among plant species in Gansu Province exhibited a high degree of spatial dependence (**Figure 9**). In the southeastern floristic region of Gansu, plant families show a strong positive co-occurrence tendency. However, as the spatial focus shifts to the northwestern region, the number of positive co-occurrences decreases, while negative co-occurrences increase. This pattern suggests that in the relatively favorable climatic conditions of southeastern Gansu, plant species are more likely to exhibit symbiotic or coexistent relationships. In contrast, in the more extreme climatic conditions of northwestern Gansu, not only does environmental filtering play a role, but interspecies competition for limited resources also intensifies, impacting community structure through competitive exclusion and reduced co-occurrence.

## 4 Discussion

### 4.1 Dominant role of environmental factors in plant distribution in Gansu

Understanding the environmental determinants of plant distribution in Gansu is crucial for predicting shifts in species ranges and community compositions, particularly in the context of global climate change (Thuiller et al., 2005). The JSDM model quantified and evaluated the influence of ecological factors on the spatial distribution of seed plants in Gansu. Analysis revealed that fixed effects explained 95.4% of the explainable variance, demonstrating that environmental factors largely determine plant distribution in the region. This supports the ecological niche theory, which posits that species distributions are primarily driven by their niche requirements and alignment with environmental conditions (Kearney, 2006). The diversity of topography and climate in Gansu provides suitable habitats for various species, leading to an environmentally driven distribution pattern (Mao et al., 2020). In Gansu, the presence of varied microclimates and diverse topographical gradients, ranging from the arid deserts of the Hexi Corridor to the forested mountains of southeastern Gansu, enhances niche availability and fosters niche differentiation (Chase et al., 2003). This environmental heterogeneity explains why the region hosts a wide variety of species despite being part of a generally arid and resource-limited landscape.

Comparisons with other arid and semi-arid regions, such as the Tibetan Plateau, the Mediterranean Basin, and the Southwestern United States, reveal similar patterns of environmentally driven plant distributions. In the Tibetan Plateau, plant community composition is shaped by climate and soil variables, where factors such as temperature and precipitation gradients create distinct vegetation zones (Körner, 2007). Similarly, studies in the Mediterranean Basin have highlighted the importance of climatic variables, particularly seasonal precipitation and temperature, in driving species diversity and distribution, with biotic interactions playing a secondary role (Thuiller et al., 2005). In the arid ecosystems of the Southwestern United States, water availability has been identified as the primary environmental filter, dictating species survival, community composition, and productivity (Huxman et al., 2004). These comparisons demonstrate that environmental filtering is a universal mechanism shaping plant distributions in resource-limited ecosystems globally.

However, Gansu’s unique combination of arid deserts, semi-arid grasslands, and forested mountains provides a distinctive mosaic of habitats compared to other regions. Unlike the largely homogeneous aridity of the Tibetan Plateau or the Mediterranean Basin’s coastal influences, Gansu’s diverse topography creates a high degree of environmental variability at relatively small spatial scales. This variability enhances microhabitat heterogeneity and facilitates the coexistence of species through spatial niche partitioning, a phenomenon also observed in other mountainous regions like the Andes and the Altai Mountains (Vetaas and Grytnes, 2002). Such spatial partitioning highlights the importance of topographical complexity in mitigating the harshness of arid conditions and promoting biodiversity.

Globally, while environmental gradients are recognized as critical drivers of species distribution and diversity, the relative influence of biotic interactions and evolutionary history varies across regions (Ricklefs, 2004). In biodiversity hotspots such as the Amazon rainforest and Southeast Asian tropical rainforests, historical evolutionary processes and high levels of biotic interactions (e.g., competition, mutualisms) contribute substantially to species distribution patterns (Rosindell et al., 2011). In these regions, random effects associated with species interactions and historical contingencies explain a larger proportion of the variance in species distributions compared to arid and semi-arid regions like Gansu. The relatively smaller influence of random effects in Gansu reflects the predominance of environmental filtering in shaping plant communities in resource-limited ecosystems, where abiotic constraints often outweigh biotic factors.

Our findings also resonate with the stress-gradient hypothesis, which suggests that the balance between competition and facilitation shifts along environmental stress gradients (Bertness and Callaway, 1994). In harsh environments like the Hexi Corridor, facilitative interactions such as those between nurse plants and dependent species likely play a crucial role in maintaining biodiversity by mitigating abiotic stress (Callaway et al., 2002). Similar phenomena have been observed in other arid regions, such as the Chihuahuan Desert and the Patagonian steppe, where nurse plants facilitate the establishment of understory species by providing shade, reducing water loss, and improving soil fertility (Holmgren et al., 1997). These facilitative interactions are essential for promoting coexistence and stabilizing plant communities under extreme environmental conditions.

From a global perspective, climate change is expected to intensify environmental filtering and shift species distributions as temperature and precipitation patterns change (Chen et al., 2011). In arid and semi-arid regions like Gansu, warming temperatures and increased aridity may exacerbate water scarcity and soil degradation, further constraining plant distributions and threatening biodiversity. The pronounced environmental filtering observed in Gansu suggests that species with narrow ecological niches or limited dispersal capabilities may be particularly vulnerable to climate change, highlighting the urgent need for conservation strategies that prioritize habitat protection and connectivity. For example, maintaining ecological corridors and restoring degraded habitats can help mitigate the impacts of climate change by facilitating species dispersal and range shifts, as demonstrated in studies from the Southwestern United States and Australia (Heller and Zavaleta, 2009).

### 4.2 Regional differences: contrasting drivers in southeast and northwest Gansu

Gansu Province’s substantial geographical and environmental heterogeneity creates a complex mosaic of habitats that influence plant distribution patterns at multiple spatial scales. Spatial gradients in elevation, temperature, precipitation, and solar radiation directly shape plant-environment interactions and generate distinct floristic regions. Findings reveal that southeast and northwest Gansu exhibit significant differences in environmental drivers and species responses, underscoring the importance of explicitly addressing environmental gradients when analyzing and interpreting plant distribution patterns.

The contrasting floristic differentiation between northwest and southeast Gansu highlights the scale-dependence of ecological processes and their influence on plant distribution patterns. In the northwest floristic region, solar radiation and mean annual precipitation were identified as the primary limiting factors, reflecting the challenges of water scarcity and high radiation pressure in arid environments (Lloret et al., 2010). These findings are consistent with global studies in arid and semi-arid regions, such as the Australian Outback, where precipitation and radiation are critical drivers of plant community structure and productivity (Wu et al., 2014). In the southeast region, solar radiation and elevation emerged as the most significant environmental factors, reflecting the influence of topography and microclimatic diversity in shaping plant communities (Zhao et al., 2022). Complex topographies, such as those found in mountainous regions, create a range of microhabitats that allow for niche differentiation and species coexistence. This pattern is mirrored in the Andes Mountains, where steep elevation gradients generate a wide variety of thermal and hydrological niches, supporting high species richness and endemism (Rahbek et al., 2019a). Similarly, in the Hengduan Mountains of southwestern China, elevation and solar radiation have been identified as key drivers of plant distribution, promoting the coexistence of diverse plant communities across fine-scale climatic gradients (Grime and Levin, 2006). This is also similar to patterns observed in tropical montane ecosystems, such as the Eastern Arc Mountains of Africa, where high energy input and topographic complexity enhance biodiversity through niche partitioning (McCain and Grytnes, 2010).

Globally, environmental filtering is the dominant mechanism in arid and resource-limited ecosystems, where species must adapt to extreme abiotic stressors, while biotic interactions become more important in regions with greater resource availability or environmental stability (Loreau et al., 2001). The contrast between northwest and southeast Gansu reflects this global trend, with abiotic constraints prevailing in the arid northwest and biotic interactions facilitating coexistence in the resource-rich southeast.

### 4.3 Key environmental drivers

Among the specific environmental factors influencing plant distribution in Gansu, habitat type has the strongest impact, explaining 33.5% of the variance. This is likely because habitat type integrates multiple ecological factors, such as soil type, topographical features, and vegetation structure, all of which directly affect plant growth and distribution (Huston, 2002; Sanchez-Mercado and Paris, 2010). Elevation (22.1%), mean annual temperature (20.3%), and mean annual precipitation (15.1%) also contribute significantly. Elevation changes are often accompanied by variations in temperature, humidity, and oxygen levels, which profoundly influence plant physiological and ecological processes (Körner, 2007). Meanwhile, temperature and precipitation, as key climatic factors, directly affect photosynthesis, water use efficiency, and plant development (Woodward, 1987; Jiao et al., 2016). Gansu’s complex geography, characterized by substantial environmental gradients including elevation, temperature, precipitation, and solar radiation, underscores the critical role of these factors in shaping plant distribution. These findings align with ecological theories emphasizing the importance of environmental gradients in determining species distributions (Wiens and Graham, 2005).

Interestingly, when considering all environmental variables together, solar radiation has a relatively minor influence on plant distribution (4.4%). However, solar radiation remains a major factor in both the northwest and southeast floristic regions. This likely reflects Gansu’s location at the transition zone of the Qinghai-Tibet Plateau, where significant regional differences in solar radiation occur. The northwest region, characterized by arid and semi-arid conditions, experiences high solar intensity and strong evapotranspiration, impacting plant water balance (Huxman et al., 2004). In the southeast region, where complex topography creates microclimatic variation in sunlight exposure, these differences in solar conditions may influence plant habitat selection and adaptability (Bennie et al., 2008). The findings resonate with the stress-gradient hypothesis, which posits that in harsh environments, environmental filtering becomes more pronounced, reducing the influence of biotic interactions on species distribution (Ziffer-Berger et al., 2014). Gansu’s regions, particularly the arid northwest, exemplify this pattern where abiotic stressors like water scarcity and high solar radiation limit species coexistence primarily through environmental constraints rather than competitive exclusion (Hobbs and Harris, 2001). As temperature and precipitation patterns alter, the established environmental filters may change, leading to potential mismatches between species’ niches and their habitats (Parmesan and Yohe, 2003).

### 4.4 Phylogenetic responses and adaptive strategies of plant families

At the family level, plants exhibit independent responses to environmental covariates, such as climate and soil factors, suggesting a diversity of adaptive strategies in response to varying environmental conditions. This phenomenon indicates that different plant families have not followed a uniform adaptive model through evolution; instead, they have developed unique ecological adaptations specific to their environmental contexts (Weigel et al., 2023).

Niche conservatism refers to the tendency of species within a lineage to retain ancestral ecological traits, with ecological niches remaining relatively stable over evolutionary time (Pyron et al., 2015). In this study, niche conservatism was not significant at the family level, as plant families’ responses to environmental factors were weakly correlated with their phylogenetic relationships. This indicates that environmental responses at a provincial scale are more likely shaped by independent evolutionary events and specific environmental selection pressures than by phylogenetic constraints. The high degree of niche plasticity at the family level suggests that ecological traits have diversified and adapted significantly over evolutionary time, with convergent evolution playing a major role in driving similar adaptive traits across unrelated families in response to comparable environmental pressures (Losos, 2008).

Analysis of the significance of species’ responses to environmental factors indicates that interactions between environmental covariates and species occurrence (i.e., species niches) do not display a pronounced phylogenetic structure. The diversity of mechanisms underlying plant community formation highlights the complexity of these processes. Different plant families may adopt entirely different adaptive strategies when faced with similar environments, suggesting that plant community structure and distribution are influenced by more than just phylogenetic relationships. Instead, these structures are shaped by a variety of environmental factors, likely encompassing independent adaptations, evolutionary innovations, and localized ecological selection pressures. However, certain closely related families, particularly within the Lamiales order, exhibit similar responses to environmental factors. This supports the view in phylogenetic ecology that closely related species may share ecological traits (Cavender-Bares et al., 2009; Wiens et al., 2010). This phylogenetic signal suggests that traits linked to phylogeny may ultimately shape species’ ecological niches, aligning with the broader understanding that many plant traits display some degree of evolutionary conservatism (Weigel et al., 2023). Thus, while taxonomic dispersion is generally high, robust phylogenetic structuring emerges within specific closely related groups, where species exhibit phylogenetically consistent responses to environmental factors. This reflects the role of phylogenetic signals in ecological adaptation within certain lineages, highlighting the interplay between evolutionary history and ecological responses (Svenning et al., 2015).

The analysis of interspecies relationships further supports this conclusion. Most plant families did not exhibit significant negative correlations, indicating that competition is not the primary factor limiting species coexistence on a macro scale. In the southeastern floristic region, positive co-occurrence among plant families was more prevalent, likely reflecting mutualistic or symbiotic interactions in resource-rich environments (Mougi, 2020). Conversely, the northwestern region exhibited higher negative co-occurrence, likely due to environmental stress and limited resources intensifying interspecies competition (Hamer et al., 2022). This indicates that species co-occurrence exhibits highly scale-dependent patterns (Weigel et al., 2023), which are consistent with the facilitation-stress gradient hypothesis (Holmgren and Scheffer, 2010). According to this hypothesis, facilitative interactions dominate under benign environmental conditions, while competitive interactions become more pronounced under stressful conditions. These findings suggest that biodiversity conservation strategies should be region-specific: in the southeast, promoting mutualistic interactions and maintaining resource-rich habitats could enhance plant diversity, while in the arid northwest, efforts should focus on mitigating environmental stressors and reducing competitive pressures to support species coexistence.

### 4.5 Conservation strategies and future research directions

This study underscores the critical role of environmental factors in shaping plant spatial distribution of seed plants in Gansu, highlighting the complex interplay between ecological adaptation and evolutionary history. The diverse responses of plant families and regions to these factors emphasize the importance of understanding the mechanisms underlying these patterns, particularly in the context of climate change (Parmesan and Yohe, 2003; Thuiller et al., 2005). In Gansu, the region’s diverse geographic environments significantly influence plant diversity, necessitating comprehensive research that integrates functional trait analyses to elucidate species’ responses to environmental gradients. Additionally, incorporating metacommunity dynamics can clarify spatial processes and dispersal limitations, providing valuable insights into shifts in species distributions and improving our understanding of plant-environment interactions (Leibold et al., 2004).

Our findings demonstrate that competition is not the primary factor limiting species coexistence. Instead, environmental filtering and adaptive strategies play a more significant role in shaping plant distributions (Chesson, 2000; Leibold et al., 2004). This insight shifts the focus of plant diversity conservation in Gansu from mitigating interspecific competition to emphasizing the preservation of diverse environmental conditions and habitat features that facilitate coexistence. In arid and semi-arid regions such as the Hexi Corridor, where abiotic stressors such as water scarcity, high solar radiation, and soil degradation dominate, conservation strategies should prioritize improving water availability, controlling soil erosion, and restoring degraded habitats. These efforts could include wetland conservation, afforestation with drought-tolerant species, and implementing water-saving irrigation techniques to enhance plant resilience in resource-limited environments. Conversely, in resource-rich southeastern regions such as the Qinling and Minshan Mountains, where mutualistic interactions are more prevalent, conservation efforts should aim to preserve biodiversity hotspots by protecting primary forests, maintaining undisturbed habitats, and fostering positive interspecific interactions, such as mutualistic networks involving pollinators and seed dispersers. Monitoring the impacts of climate change in these regions is also critical to ensure conservation measures are adaptive to shifting species distributions and ecological conditions.

To establish conservation priorities, it is essential to consider environmental gradients, interspecific relationships, and species vulnerabilities to climate change and human disturbances. For example, in transitional zones between arid and semi-arid regions, where environmental heterogeneity and biodiversity significance are high, conservation efforts should focus on protecting habitats with high ecological importance (Thuiller et al., 2005). Conservation strategies should also emphasize species and habitats at greater risk of extinction or degradation due to climate change or anthropogenic impacts (Brook et al., 2008). Incorporating spatial modeling techniques to map species richness, functional diversity, and ecosystem services can help guide the efficient allocation of conservation resources (Wisz et al., 2013b). Furthermore, integrating assessments of interspecific relationships into conservation planning can identify key species that facilitate coexistence and enhance community stability, thereby maximizing biodiversity conservation outcomes.

While this study focuses on plant distributions in Gansu, the findings have broader implications for understanding plant distribution patterns in other regions and ecosystems. Similar studies in arid and semi-arid regions, such as the Mediterranean Basin or the Southwestern United States, have demonstrated that environmental gradients and ecological filtering are critical drivers of plant distribution (Maestre et al., 2005; Zhang and Tielbörger, 2020). However, the specific factors and their relative contributions are highly context-dependent, shaped by the unique climatic, geological, and ecological characteristics of each region. For instance, the role of mutualistic interactions, identified as a key factor in southeastern Gansu, may vary in ecosystems where biotic interactions are less prominent. Similarly, the importance of abiotic stressors such as drought and soil degradation, observed in the Hexi Corridor, may be less pronounced in regions with more stable climatic conditions. Therefore, although the overarching principles identified in this study, including environmental filtering, adaptive strategies, and positive interspecific interactions, have broad applicability, specific conservation strategies should be adapted to the unique ecological and environmental contexts of each locality.

Based on the inadequacy of existing plant functional trait data, future conservation planning based on functional traits will be needed to identify species or groups of plants with critical ecological roles and adaptive capacity. For instance, prioritizing the protection of plants with traits that enhance drought tolerance in arid regions or shade tolerance in forest ecosystems can help maintain the functional stability of plant communities. Conservation strategies should also consider the role of facilitator species, such as nurse plants in deserts or dominant tree species in forests, which promote coexistence and stability within plant communities (Michalet et al., 2006; Callaway, 2007).

Despite the robust insights provided by this study, several methodological limitations should be acknowledged. Multicollinearity among environmental variables remains a challenge, as it can obscure the individual contributions of predictors. Pairwise correlations were evaluated in this study to mitigate collinearity. Overfitting, particularly in complex models, also poses a limitation, which was addressed through cross-validation techniques to improve the generalizability of the model. Additionally, reliance on species distribution databases introduces potential biases, as these databases often contain incomplete records or sampling discrepancies. Intensive sampling in certain areas may inflate estimates of species richness, while under-sampled regions may underestimate diversity. To address these limitations, future research should incorporate field surveys to validate and complement existing records, use occupancy modeling techniques to account for detection biases, and include uncertainty estimates to improve data reliability and robustness.

Future research should further explore the ecological mechanisms underlying species responses to environmental gradients and interspecific relationships, particularly through field-based studies and advanced modeling approaches. Such efforts would provide stronger empirical support for the development of conservation strategies that align with the ecological realities of Gansu Province. By addressing these aspects, this study aims to advance scientific understanding while informing practical measures for biodiversity conservation and ecosystem management, ensuring that conservation initiatives are both scientifically sound and regionally applicable.

## 5 Conclusion

This study employed JSDM to investigate the spatial distribution patterns and influencing factors of plant communities in Gansu Province. The findings reveal that environmental factors are the predominant determinants of plant spatial patterns. Key environmental variables, including habitat type, elevation, mean annual temperature, and precipitation, were identified as major drivers of plant distribution, while solar radiation played a region-specific role, particularly in the northwest and southeast floristic regions. The analysis also demonstrated that plant family responses to environmental covariates are largely independent and weakly correlated with phylogenetic relationships, indicating minimal niche conservatism at the family level. However, certain closely related families within the order Lamiales exhibited similar responses to environmental factors, suggesting that phylogenetic signals can influence ecological adaptations within specific lineages. The study further revealed that interspecies competition is not the primary constraint on species coexistence at a macro scale, with positive co-occurrence patterns in resource-rich southeastern regions and increased negative co-occurrence in the resource-limited northwest. These findings highlight the combined influence of environmental factors, evolutionary history, and interspecies relationships in shaping plant distribution and community structure. These insights have significant implications for biodiversity conservation and ecosystem management, emphasizing the need for region-specific strategies that consider both environmental dependencies and species interactions.

In conclusion, this study underscores the critical role of environmental gradients in shaping plant community structures and spatial patterns in Gansu Province. The findings highlight the diverse adaptive strategies employed by plant families in response to environmental changes and emphasize the importance of region-specific conservation strategies that account for both environmental dependencies and species interactions. By addressing the methodological limitations outlined in this study and incorporating field-based data, future research can refine our understanding of plant-environment dynamics and contribute to more effective biodiversity conservation strategies.

## Data Availability

The original contributions presented in the study are included in the article/**Supplementary Material**. Further inquiries can be directed to the corresponding authors.
