# Supplementary material for: Environmental response strategies for the spatial distribution of seed plants in Gansu
Source: Front Plant Sci. 2025 Feb 17;16:1526269. doi: 10.3389/fpls.2025.1526269 (PMC11872896; doi:10.3389/fpls.2025.1526269)
Supplement: Supplementary file 1 [file DataSheet1.docx]

**Supplementary information**


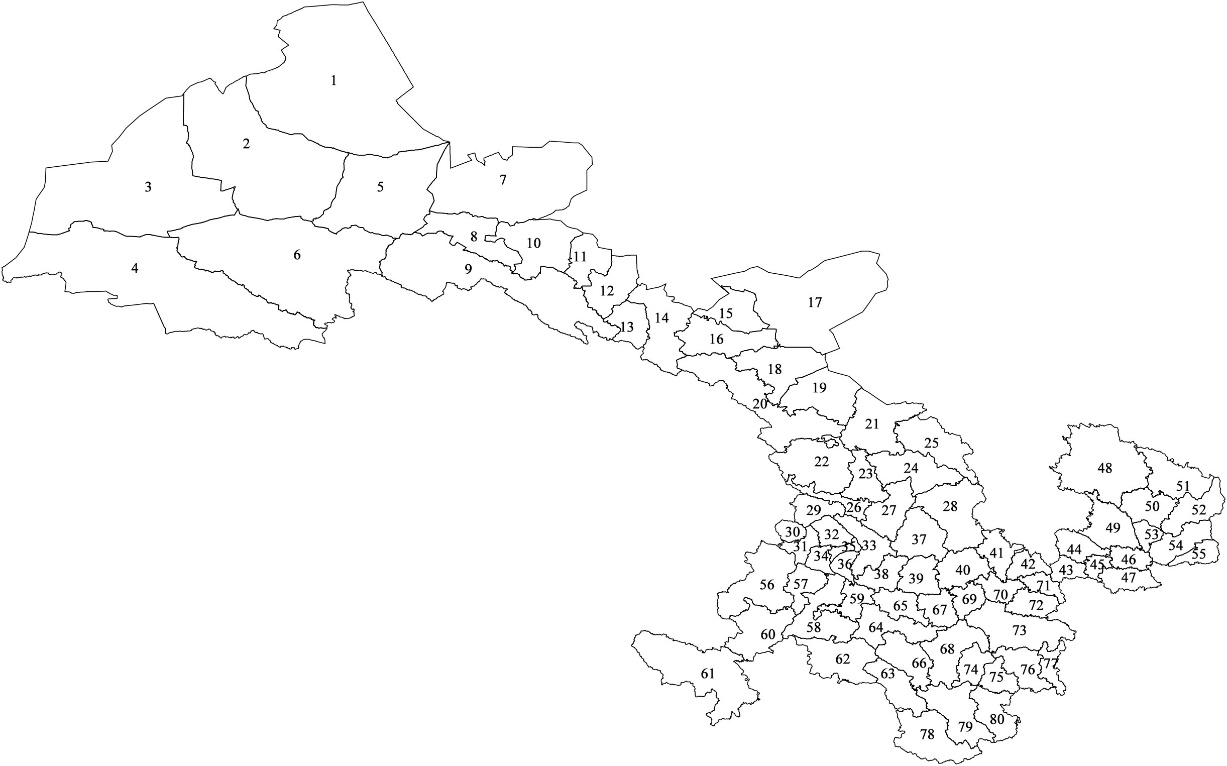


**Fig. S1** Gansu County Grid Cell. The numbers correspond with the code in Supplementary Table S1, which indicate name of each county. The map was generated using ArcGIS 10.8.

**Table S1.** Area and floristic regions for each county in Gansu. Code corresponds with the numbers in map of administrative counties (Supplementary Figure S1), which show the position of each county in Gansu.

| **Code** | **County** | **Area(km^2^)** | **Floristic region** |
| --- | --- | --- | --- |
| 1 | North-Subei | 38000 | Ⅱ |
| 2 | Guazhou | 24100 | Ⅱ |
| 3 | Dunhuang | 31200 | Ⅱ |
| 4 | Akesai | 32374 | Ⅰ |
| 5 | Yumen | 13389 | Ⅱ |
| 6 | South-Subei | 28748 | Ⅰ |
| 7 | Jinta | 18800 | Ⅱ |
| 8 | Suzhou | 6321 | Ⅱ |
| 9 | Sunan | 14780 | Ⅰ |
| 10 | Gaotai | 6016 | Ⅱ |
| 11 | Linze | 2729 | Ⅱ |
| 12 | Ganzhou | 4240 | Ⅱ |
| 13 | Minle | 3687 | Ⅰ |
| 14 | Shandan | 9374 | Ⅰ |
| 15 | Yongchang | 5867 | Ⅱ |
| 16 | Jinchuan | 3060 | Ⅱ |
| 17 | Minqin | 15907 | Ⅱ |
| 18 | Liangzhou | 5081 | Ⅱ |
| 19 | Gulang | 5103 | Ⅰ |
| 20 | Tianzhu | 7147 | Ⅰ |
| 21 | Jingtai | 5483 | Ⅲ |
| 22 | Yongdeng | 5652 | Ⅲ |
| 23 | Gaolan | 2556 | Ⅲ |
| 24 | Baiyin | 4534.2 | Ⅲ |
| 25 | Jingyuan | 4754.8 | Ⅲ |
| 26 | Lanzhou | 1630 | Ⅲ |
| 27 | Yuzhong | 3245 | Ⅲ |
| 28 | Huining | 6439 | Ⅲ |
| 29 | Yongjing | 1864 | Ⅳ |
| 30 | Jishishan | 910 | Ⅳ |
| 31 | Linxia | 1302 | Ⅳ |
| 32 | Dongxiang | 1512 | Ⅳ |
| 33 | Lintao | 2851 | Ⅳ |
| 34 | Hezheng | 960 | Ⅳ |
| 35 | Guanghe | 538 | Ⅳ |
| 36 | Kangle | 1083 | Ⅳ |
| 37 | Anding | 4225 | Ⅲ |
| 38 | Weiyuan | 2034 | Ⅳ |
| 39 | Longxi | 2657 | Ⅳ |
| 40 | Tongwei | 2899 | Ⅳ |
| 41 | Jingning | 2193 | Ⅴ |
| 42 | Zhuanglang | 1558 | Ⅴ |
| 43 | Huating | 1183 | Ⅸ |
| 44 | Kongtong | 1936 | Ⅸ |
| 45 | Chongxin | 852 | Ⅴ |
| 46 | Jingchuan | 1409 | Ⅴ |
| 47 | Lingtai | 2038 | Ⅴ |
| 48 | Huanxian | 9236 | Ⅴ |
| 49 | Zhenyuan | 3500 | Ⅴ |
| 50 | Qingcheng | 2673 | Ⅴ |
| 51 | Huachi | 3776 | Ⅴ |
| 52 | Heshui | 2976 | Ⅴ |
| 53 | Xifeng | 996 | Ⅴ |
| 54 | Ningxian | 2633 | Ⅴ |
| 55 | Zhengning | 1329 | Ⅴ |
| 56 | Xiahe | 6266 | Ⅷ |
| 57 | Hezuo | 2670 | Ⅶ |
| 58 | Zhuoni | 4920 | Ⅶ |
| 59 | Lintan | 2057 | Ⅶ |
| 60 | Luqu | 4260 | Ⅷ |
| 61 | Maqu | 10190 | Ⅷ |
| 62 | Diebu | 5148 | Ⅶ |
| 63 | Zhouqu | 3010 | Ⅶ |
| 64 | Minxian | 3500 | Ⅶ |
| 65 | Zhangxian | 2164 | Ⅵ |
| 66 | Tanchang | 3315 | Ⅸ |
| 67 | Wushan | 2011 | Ⅵ |
| 68 | Lixian | 4264 | Ⅵ |
| 69 | Gangu | 1573 | Ⅵ |
| 70 | Qinan | 1602 | Ⅵ |
| 71 | Zhangjiachuan | 1311 | Ⅵ |
| 72 | Qingshui | 2012 | Ⅵ |
| 73 | Tianshui | 5922 | Ⅵ |
| 74 | Xihe | 1862 | Ⅵ |
| 75 | Chengxian | 1678 | Ⅸ |
| 76 | Huixian | 2699 | Ⅵ |
| 77 | Liangdang | 1408 | Ⅵ |
| 78 | Wenxian | 5002 | Ⅸ |
| 79 | Wudu | 4642 | Ⅸ |
| 80 | Kangxian | 2968 | Ⅸ |


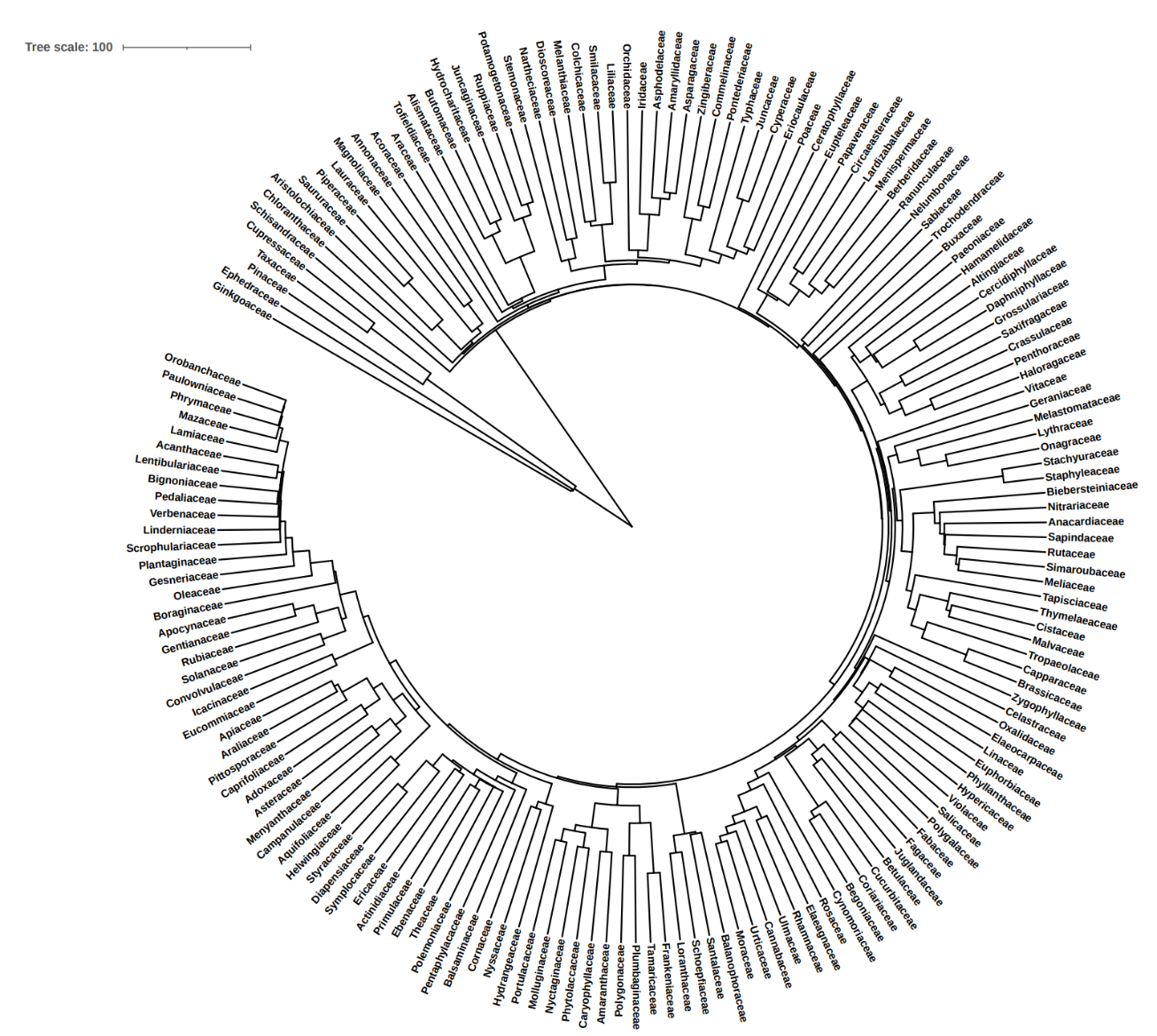


**Fig. S2** Phylogenetic tree of seed plant families in Gansu


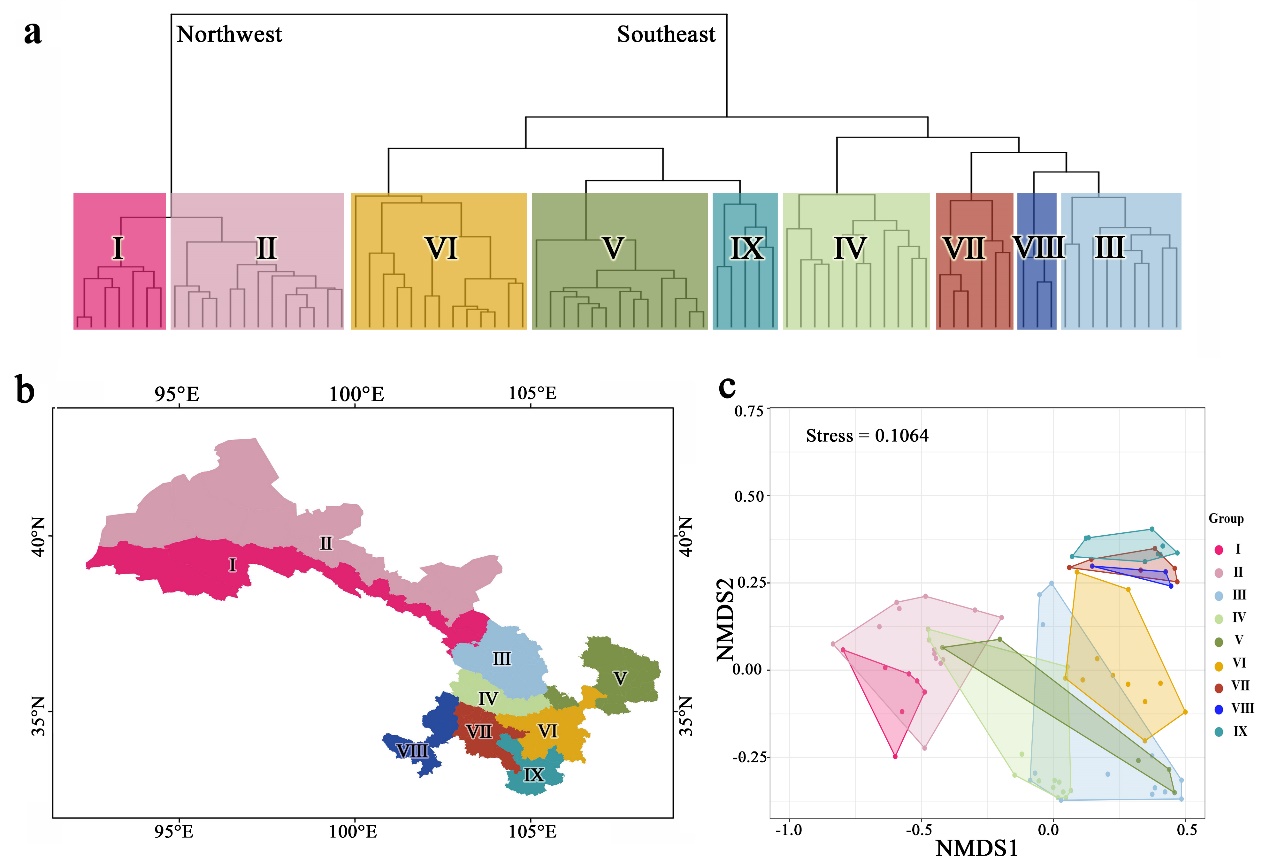


**Fig.S3** Dendrogram (**a**) and map (**b**) resulting from UPGMA hierarchical clustering and the NMDS ordination of grid cell assemblages based on phylogenetic beta diversity distance matrixes at the genus level (**c**). The dendrogram highlights the nine phytogeographical regions, which are also represented by corresponding colors in the NMDS ordination. Additionally, the map, generated using ArcGIS 10.8, visually displays the spatial distribution of these regions. I—the northern foothills of the Qilian Mountains; Ⅱ—the hinterland of the Hexi Corridor; Ⅲ—the Lanzhou–Baiyin wilderness region; Ⅳ—the Loess Plateau in the central region; V—the Loess Plateau in the east region; Ⅵ—the western Qinling Mountains; Ⅶ—the transitional zone from Gannan Plateau to Longnan Mountainous Region. Ⅷ—the Gannan Plateau; and Ⅸ—the Longnan Mountainous Region (Li et al., 2023).
